# Correlation of gut microbiota with leukopenia after chemotherapy in patients with colorectal cancer

**DOI:** 10.1186/s12866-023-03067-6

**Published:** 2023-11-17

**Authors:** Ni Xiaofeng, Chu Jian, Wang Jingjing, Qu Zhanbo, Song Yifei, Zhuang Jing, Han Shuwen

**Affiliations:** 1 Huzhou Central Hospital, Affiliated Central Hospital HuZhou University, Huzhou, China; 2 Fifth School of Clinical Medicine of Zhejiang, Huzhou Central Hospital, Chinese Medical University, Huzhou, China; 3 Key Laboratory of Multiomics Research and Clinical Transformation of Digestive Cancer of Huzhou, Huzhou, China

**Keywords:** Colorectal cancer, Myelosuppression, Chemotherapy, Gut microbiota, Leukopenia

## Abstract

**Background:**

The most common toxic side effect after chemotherapy, one of the main treatments for colorectal cancer (CRC), is myelosuppression.

**Objective:**

To analyze the correlation between gut microbiota and leukopenia after chemotherapy in CRC patients.

**Methods:**

Stool samples were collected from 56 healthy individuals and 55 CRC patients. According to the leukocytes levels in peripheral blood, the CRC patients were divided into hypoleukocytes group (*n* = 13) and normal leukocytes group (*n* = 42). Shannon index, Simpson index, Ace index, Chao index and Coverage index were used to analyze the diversity of gut microbiota. LDA and Student's t-test(St test) were used for analysis of differences. Six machine learning algorithms, including logistic regression (LR) algorithm, random forest (RF) algorithm, neural network (NN) algorithm, support vector machine (SVM) algorithm, catboost algorithm and gradient boosting tree algorithm, were used to construct the prediction model of gut microbiota with leukopenia after chemotherapy for CRC.

**Results:**

Compared with healthy group, the microbiota alpha diversity of CRC patients was significantly decreased (*p* < 0.05). After analyzing the gut microbiota differences of the two groups, 15 differential bacteria, such as *Bacteroides, Faecalibacterium* and *Streptococcus,* were screened. RF prediction model had the highest accuracy, and the gut microbiota with the highest predictive value were *Peptostreptococcus, Faecalibacterium, and norank_f__Ruminococcaceae, respectively*. Compared with normal leukocytes group, the microbiota alpha diversity of hypoleukocytes group was significantly decreased (*p* < 0.05). The proportion of *Escherichia-Shigella* was significantly decreased in the hypoleukocytes group. After analyzing the gut microbiota differences of the two groups, 9 differential bacteria, such as *Escherichia-Shigella, Fusicatenibacter and Cetobacterium,* were screened. RF prediction model had the highest accuracy, and the gut microbiota with the highest predictive value were *Fusicatenibacte, Cetobacterium, and Paraeggerthella*.

**Conclusion:**

Gut microbiota is related to leukopenia after chemotherapy. The gut microbiota may provide a novel method for predicting myelosuppression after chemotherapy in CRC patients.

**Supplementary Information:**

The online version contains supplementary material available at 10.1186/s12866-023-03067-6.

## Introduction

Colorectal cancer (CRC) is a common malignant tumor, and its incidence and mortality rates have been on the rise in the past 10 years [1]. The number of new CRC cases worldwide is expected to increase to 2.5 million by 2035, which will surpass common cancers such as liver and stomach cancers [2]. Studies have proved that approximately 90% of CRC occurs sporadically, and the remainder was caused by genetic factors or exposure to specific environmental factors [3]. CRC is the result of a synergistic effect of environmental [4], nutritional [5], and genetic [6] factors, and it has been shown that gut microbiota [7, 8] are involved in the development and progression of CRC.

Gut microbiota is important participant in human metabolism, which produces fatty acids and other substances that promote the growth and differentiation of human epithelial cells and are involved in the synthesis of vitamins and the absorption of various ions [9]. The mechanisms by which gut microbiota affects carcinogenesis, inflammation, and immune and therapeutic responses at the local level have been revealed in existing studies [10, 11]. There is growing evidence for a direct pathogenic role of the gut microbiota in regulating signaling pathways, antitumor immune responses, and cell proliferation [12]. Normal gut microbiota plays an important role in the homeostasis of the intestinal environment, including involvement in the protection, structure formation and metabolism of the intestinal epithelium [13]. Imbalance of gut microbiota alters the intestinal microenvironment, including changes in intestinal epithelial genes, development of inflammatory responses, production of toxic metabolites, and damage to the intestinal epithelial barrier [14]. All these changes are potential pathogenic factors for CRC [15]. In addition, *Bifidobacterium* is one of the most commonly used probiotics with beneficial effects on various diseases, including CRC [16]. However, the more specific role of gut microbiota in the development of CRC remains shrouded in mystery.

Chemotherapy, which is significantly associated with gut microbiota [17], is an important treatment for CRC. After chemotherapy, the relative abundance of the phylum *Bacteroides* significantly decreased, while the relative abundance of the families *Clostridiaceae* and *Streptococcaceae* increased [18]. Moreover, chemotherapy drug treatment may affect the normal organ functions of body [19]. The most concerned side effect after chemotherapy is myelosuppression that is mainly due to the cytotoxic effects of chemotherapeutic agents [20]. Myelosuppression may manifest as white blood cell(WBC) or neutropenia, thrombocytopenia, or even anemia. When body is invaded by external bacteria and viruses, the WBC will respond quickly to protect body's health by engulfing these viruses and bacteria. Therefore, the reduction of white blood cells may affect the community structure of gut microbiota [21]. In addition, gut microbiota may also in turn affect the number of white blood cells in the blood through hematopoietic function [22]. It is inferred that there may be a correlation between gut microbiota and leukopenia after chemotherapy.

The study focused on using microbial sequencing technology to analyze the diversity and community structure of gut microbiota with leukopenia after chemotherapy in CRC patients. The differential gut microbiota was screened by St test, and the differential gut microbiota was used to establish the prediction model of leukopenia. Gut microbiota can provide a potential research direction for the prevention and treatment leukopenia after chemotherapy in patients with CRC.

## Methods

### Subjects

From February 2019 to May 2021, the participants were 56 healthy volunteers from the Physical Examination Center of Huzhou Central Hospital and 55 CRC patients after chemotherapy in the Oncology Department. According to the leukocytes levels, the CRC patients were divided into hypoleukocytes group (*n* = 13) and normal leukocytes group (*n* = 42). Hypoleukocytes group and normal leukocytes group represent ranges of leukocytes values of below 3.5 × 10^9^/L and (3.5–9.5) × 10^9^/L, respectively. The general conditions of the patients and healthy individuals were shown in Tables 1 and 2.

**Table 1:** Clinical information on patients with CRC and healthy

			Healthy group	CRC group	*P*- value
Cases,n		56		55	
Sex	Male	10		32	0.001
	Female	46		23	
Hypertension	Have	13		22	0.067
	No	43		34	
Diabetes	Have	5		5	0.976
	No	51		50	
Smoking	Have	0		9	0.002
	No	56		46	
Drinking	Have	0		11	0.001
	No	56		44	
Age		50.14 ± 13.83		67.09 ± 10.38	0.001
Weight		67.09 ± 10.38		58.29 ± 12.41	0.232
Height		160.72 ± 6.6		163.05 ± 9.34	0.141
RBC(10^12/L)		4.56 ± 0.44		4.82 ± 6.26	0.765
Hb(g/L)		135.96 ± 17.01		122.93 ± 20.03	0.001

**Table 2:** Clinical basic information of colorectal cancer patients with low WBC group and normal WBC group after CRC chemotherapy

		Low WBC group	Normal WBC group	*P*- value
Cases,n		13	42	
Sex	Male	7	25	0.533
	Female	6	17	
Age		65.07 ± 11.32	67.76 ± 10.1	0.406
Stage	I	0	0	0.213
	II	11	32	
	III	2	8	
	IV	0	2	
Cr(μmol/L)		69.68 ± 13.58	73.94 ± 20.01	0.462
RBC(10^12/L)		3.56 ± 0.56	5.23 ± 7.19	0.390
Hb(g/L)		108.5 ± 19.39	127.74 ± 18	0.001
Albumin(g/L)		38.00 ± 5.10	37.78 ± 4.14	0.874
TG(mmol/L)		1.08 ± 0.47	1.50 ± 0.75	0.093
TC(mmol/L)		3.96 ± 0.94	4.50 ± 0.89	0.098
HDL(mmol/L)		42.45 ± 11.79	47.07 ± 14.79	0.364
LDL(mmol/L)		86.53 ± 30.45	104.66 ± 34.13	0.300
Chemotherapy regimen	FOLFOX	13	42	-

### Inclusion criteria

The control group: They had no respiratory diseases, gastrointestinal diseases, oral diseases, malignant tumors, and tumor-related symptoms in the past two years.

The CRC group: 1) CRC was confirmed by pathological examination. 2) The predicted survival time of CRC patients was ≥ 3 months. 3) CRC patients did chemotherapy for the first time. 4) The electrocardiogram, liver and kidney function and blood routine examination of CRC patients were normal before chemotherapy. 5) Clinical staging followed the American Joint Committee on Cancer (AJCC) staging guidelines.

### Exclusion criteria

1) The blood picture of CRC patients before chemotherapy suggested myelosuppression. 2) Patients with CRC had a history of chemotherapy. 3) CRC patients were complicated with other malignant tumors. 4) Patients with CRC had a history of oral gut microbiota preparation one month before admission. 5) Patients had other intestinal diseases. 6) Chemotherapy patients with combined targeted therapy.

The Ethics Committee of Huzhou Central Hospital approved the patients’ clinical protocol and informed consent. All subjects signed informed consent in accordance with the guidelines approved by the Ethics Committee of Huzhou Central Hospital (20,201,106–02) and Chinese clinical trial registry (http://www.chictr.org.cn, No. ChiCTR1800018908).

### Fecal sample collection

Basic information and post-chemotherapy white blood cell count were obtained from the case management system of Huzhou Central Hospital with the informed consent of the patients. Stool samples were collected separately before breakfast. Approximately 5–10 g of stool samples were taken after defecation without the use of laxatives or lubricants. Within half an hour, the stool samples were stored in an ultra-low temperature refrigerator and samples were kept for no longer than 1 month.

### Gut microbiota 16S rRNA detection

- 1) DNA extraction: The total DNA was extracted from stool samples using a DNA kit according to the manufacturer's protocol. Polymerase chain reaction (PCR) was used to amplify the V3-V4 region of bacteria 16S rRNA gene.
- 2) PCR amplification: The PCR products of the same sample were mixed and detected by 2% of agarose gel electrophoresis. An AxyPrep DNA Gel recovery Kit (AxyPrep Biosciences, Union City, CA) was used to cut the gel and recover the PCR products, and Tris_HCl elution was performed with 2% of agarose electrophoresis. The PCR products were detected and quantified using the QuantiFluor™-ST blue fluorescence quantification system based on the preliminary quantitative results of electrophoresis and then mixed in the appropriate proportion according to the sequencing volume requirements of each sample.
- 3) MiSeq library construction and sequencing: One end of the DNA fragment was complementary to the primer base and fixed on the chip, the fixed base sequence on the chip was used as the primer for PCR synthesis, and the target DNA fragment to be tested was synthesized on the chip. The other end of the DNA fragment on the chip was randomly complementary to another primer nearby and also fixed, thus forming a "bridge", and PCR amplification produces DNA clusters. The DNA amplicons were linearized to become single-stranded. A modified DNA polymerase and dNTP with four fluorescent labels were added to synthesize one base per cycle. The above methods were referred to a published literature [23].

### Sequencing data bioinformatics analysis

1. The raw data were spliced for quality control, optimized for data, differentiated for samples, and then subjected to OTU clustering analysis and species taxonomy analysis, and a variety of diversity indices can be analyzed based on OTU. Based on the above analysis, a series of in-depth statistical and visualization analyses, such as multivariate analysis and difference significance test, can be performed on the community composition and phylogenetic information of multiple species.
2. Community composition analysis
  The community composition of each sample was determined at the genus level and represented by a community histogram. The abundance and diversity of microbial communities were reflected by diversity of single samples analysis, including a series of statistical analysis indices to estimate the species abundance and diversity of environmental communities. V-enn plots were used to analyze the number of coexisting and unique colonies of gut microbiota.
3. Alpha diversity analysis
  To study the microbial diversity of the fecal microbial community ecology of the sample, the diversity analysis of a single sample (Alpha diversity) could reflect the abundance and diversity of the microbial community, including a series of statistical analysis indices to estimate the species abundance and diversity of the environmental community. Mothur software (https://www.mothur.org/wiki/Download_mothur) was used to calculate the Chao abundance index, Ace index, Shannon index and Simpson index.
4. Species difference analysis
  Based on the obtained community abundance data, species difference analysis was performed using relevant analytical methods to detect differences in abundance exhibited by different groups of microbial communities. Firstly, non-parametricfactorial Kruskal–Wallis (KW) sum-rank test (nonparametricfactorial KW rank-sum test) was used to detect features with significant abundance differences and find taxa with significant differences in abundance. Subsequently, LEfSe used linear discriminant analysis (LDA) to estimate the magnitude of the effects of each component species abundance on the difference effect.

### Correlation analysis

Tutools Platform software (http://www.cloudtutu.com), a free online data analysis website, was used to draw intragroup correlation heatmaps.

### Construction of a prediction model for leukopenia after chemotherapy in patients with CRC

Logistic regression (LR), random forest (RF), neural network (NN), support vector machine (SVM), gradient boosting decision tree (GBDT) and CatBoost models were used to screen differential bacteria as construction elements. Through the decision tree classifier, the final classification was made after comprehensively considering all the results. The probability mean was used for regression analysis to select the most important gut microbiota in the sample classification. Sensitivity and specificity were calculated by setting different cut-off values. ROC curve was drawn with sensitivity as the ordinate and specificity as the abscissa, and the area under the curve (AUC) was calculated.

### Statistical analysis

For continuous variables, an independent t test was applied. For categorical variables between groups, Pearson’s chi-square or Fisher's exact test was used, depending on assumption validity. Statistical analysis was performed using SPSS V25.0 (SPSS Inc., Chicago, IL). GraphPad Prism version 8.0 (San Diego, CA) and the Tutools platform (http://www.cloudtutu.com) were used for the preparation of graphs. All tests of significance were two-sided, and p < 0.05 or corrected *p* < 0.05 was considered statistically significant.

## Results

### Comparative analysis of CRC and healthy group

#### Gut microbiota community structure and diversity in CRC

**Fig. 1:**
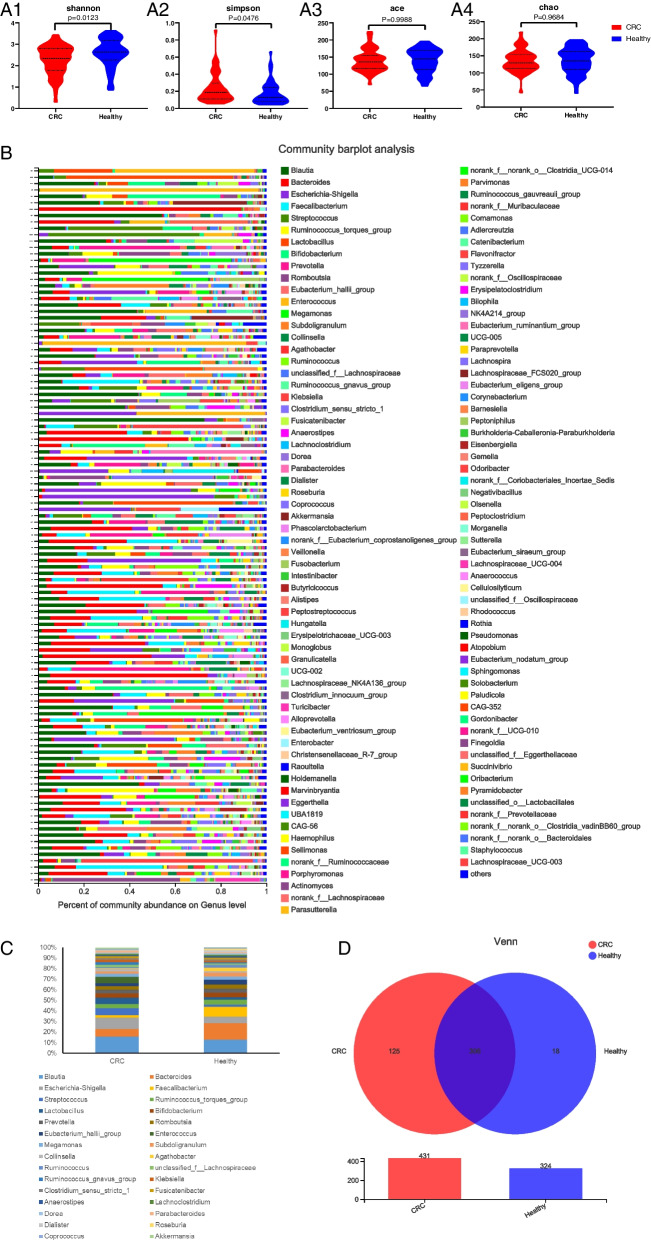
Descriptive analysis between CRC and healthy groups. **A** Alpha diversity was used to show species abundance of gut bacteria, with A1, A2, A3 and A4 showing Shannon index, Simpson index, Ace index and Chao index, respectively. **B** The gut bacteria composition between the groups was plotted. The ordinate is the group name, and the abscissa represents the proportion of bacteria in the sample. Different colors represent different bacterial groups, and the length of the column represents the proportion of bacterial groups. **C** Histograms of the cumulative percentages of the top 30 most abundant bacteria in both groups were plotted. **D** Red represents the CRC group, blue marks the healthy group, and the overlap is the number of common bacteria in the two groups. The following is a Venn diagram of the total gut bacteria at the genus level for both groups

Compared with the healthy group, the gut microbiota community diversity of CRC patients was significantly decreased (*p* < 0.05). There was no difference about the abundance in both groups (*p* > 0.05) (Fig. 1 A1–A4). The gut microbiota community structure was different between the two groups, and the bacteria was widely distributed and the number of bacteria varied (Fig. 1B). The top five bacteria with the highest composition ratio in both groups were *Blautia, Escherichia-Shigela, Streptococcus, Bacteroides* and *Faecalibacterium* (Fig. 1C). The Venn diagram showed 306 common bacteria for both groups, with 18 unique bacteria to the healthy group and 125 unique bacteria to the CRC group (Fig. 1D). The sequencing depth was shown in Table 3.

**Table 3:** Diversity index table about CRC and healthy group. chao and ace were the indices of community richness. shannon index, simpson index and coverage index were the indices of community diversity

Groups	shannon	simpson	ace	chao	coverage
CRC Group	2.892	0.101	150.101	142.667	0.999
	1.631	0.327	177.618	132.000	0.999
	2.489	0.145	118.538	117.583	1.000
	2.613	0.113	117.006	111.176	1.000
	1.429	0.460	136.888	129.882	0.999
	2.926	0.130	187.244	199.273	0.999
	1.641	0.412	93.555	92.000	1.000
	2.844	0.094	204.636	185.500	0.999
	2.719	0.101	102.252	98.231	1.000
	2.767	0.121	135.213	133.273	1.000
	1.879	0.290	171.260	141.882	0.999
	2.704	0.098	115.230	109.059	1.000
	1.837	0.277	111.264	125.429	1.000
	1.316	0.358	96.575	100.857	1.000
	1.158	0.443	69.793	42.429	0.998
	0.760	0.499	108.416	102.600	0.999
	2.407	0.218	148.816	144.438	0.999
	2.927	0.137	177.487	183.000	1.000
	2.539	0.142	140.536	146.111	1.000
	2.318	0.184	125.058	124.100	0.999
	1.706	0.432	103.358	109.667	1.000
	2.328	0.222	132.955	126.833	1.000
	2.726	0.103	130.754	128.615	1.000
	1.348	0.544	143.298	145.250	0.999
	2.517	0.121	116.788	113.067	1.000
	1.789	0.256	217.289	183.048	0.999
	1.215	0.542	121.735	97.250	1.000
	2.804	0.098	111.492	113.667	1.000
	2.316	0.236	154.455	152.500	0.999
	0.302	0.916	131.685	98.500	0.999
	1.691	0.343	144.100	139.200	1.000
	1.951	0.201	149.006	155.200	0.999
	2.894	0.144	155.083	151.563	1.000
	3.364	0.056	180.364	186.000	0.999
	2.272	0.210	122.481	121.111	1.000
	2.990	0.088	148.048	152.111	1.000
	1.534	0.268	115.092	124.100	0.999
	3.112	0.063	138.870	141.100	1.000
	3.089	0.085	224.456	219.500	0.999
	2.304	0.179	124.050	118.769	0.999
	2.134	0.253	152.615	151.500	1.000
	2.799	0.115	170.609	163.556	0.999
	2.352	0.160	118.794	122.333	0.999
	2.229	0.194	116.374	113.545	1.000
	0.496	0.800	41.150	39.200	1.000
	2.007	0.312	124.235	156.200	0.999
	1.603	0.425	107.759	112.857	1.000
	2.046	0.190	148.378	122.000	1.000
	1.193	0.578	113.560	115.100	1.000
	1.772	0.277	150.280	129.083	0.999
	3.435	0.073	177.425	180.000	0.999
	2.362	0.189	148.510	146.688	1.000
	2.053	0.225	182.335	144.750	0.999
	2.863	0.098	149.121	157.111	0.999
	0.560	0.796	138.563	123.000	0.999
	2.677	0.149	174.768	163.550	0.999
Healthy Group	2.566	0.171	113.081	116.429	1.000
	2.602	0.139	168.193	142.000	0.999
	2.017	0.275	112.717	117.100	0.999
	1.894	0.291	114.707	123.000	0.999
	1.653	0.356	67.323	68.750	1.000
	3.270	0.069	170.505	166.158	1.000
	2.328	0.186	106.361	102.375	1.000
	2.615	0.125	158.754	130.000	0.999
	2.051	0.299	113.097	112.100	1.000
	1.419	0.486	114.729	113.462	0.999
	2.465	0.119	89.719	87.091	1.000
	2.534	0.175	157.374	161.909	0.999
	2.685	0.139	137.242	143.000	1.000
	2.115	0.231	138.906	108.667	1.000
	2.232	0.168	86.454	90.600	1.000
	3.485	0.048	152.398	150.333	1.000
	2.951	0.123	167.679	170.000	0.999
	1.192	0.574	91.052	87.500	1.000
	2.055	0.291	106.252	110.167	1.000
	2.235	0.191	106.474	100.250	1.000
	2.783	0.084	167.005	129.955	0.999
	2.255	0.192	99.056	95.800	1.000
	3.199	0.081	144.829	144.091	1.000
	2.144	0.164	109.039	114.250	0.999
	2.521	0.149	132.436	126.000	1.000
	2.730	0.100	139.849	139.214	0.999
	3.291	0.065	163.416	162.250	0.999
	2.704	0.113	148.276	129.111	0.999
	2.555	0.181	153.044	150.769	1.000
	2.321	0.138	115.692	119.000	0.999
	2.542	0.160	126.846	136.143	1.000
	3.045	0.072	124.727	115.429	1.000
	2.870	0.137	150.972	155.100	1.000
	2.680	0.095	104.747	108.429	1.000
	3.184	0.072	162.511	169.000	0.999
	2.644	0.123	151.305	149.071	0.999
	2.661	0.116	119.042	127.250	1.000
	2.661	0.171	125.715	123.909	1.000
	1.995	0.294	121.669	107.077	1.000
	2.267	0.161	126.987	111.000	1.000
	2.711	0.105	94.497	93.500	1.000
	3.436	0.060	150.931	150.000	1.000
	3.086	0.087	137.683	138.667	1.000
	3.388	0.058	163.637	167.000	0.999
	2.952	0.086	212.282	220.000	0.999
	1.940	0.266	64.782	65.600	1.000
	2.862	0.106	120.150	122.143	1.000
	3.416	0.054	157.703	156.462	1.000
	2.875	0.104	130.530	115.125	1.000
	3.083	0.080	166.625	179.111	0.999
	2.285	0.211	83.214	81.143	1.000
	2.924	0.097	147.305	124.125	1.000
	2.075	0.246	153.762	150.667	0.999
	2.882	0.104	129.073	129.333	1.000
	2.817	0.111	155.504	133.000	0.999
	2.359	0.149	106.415	104.667	1.000

#### Difference of gut microbiota between CRC and healthy individuals

**Fig. 2:**
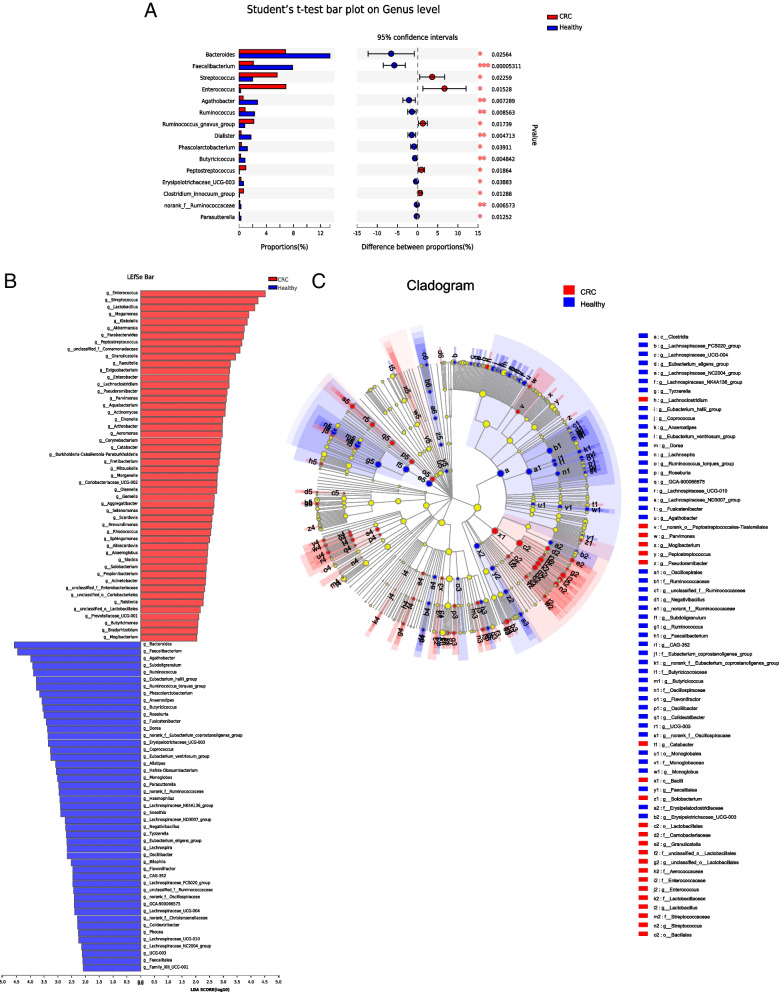
Difference analysis between CRC and healthy groups **A**: The t-test method was used to test the hypothesis of the gut microbiota of the two groups and evaluate the significance level of the difference in the abundance of the bacteria. *P* < 0.05 was considered statistically significant. **B**: LDA was used to draw histograms and count the gut microbiota with significant differences between the two groups. LDA scores were obtained using linear regression analysis. The greater the score, the greater the influence of gut microbiota abundance on differential effects. **C**: From inner circle to outer circle, the bacteria at different levels of phylum, class, order, family, genus, and species are represented in turn. Different colored nodes indicate the degree of enrichment of bacteria in the corresponding group and whether they have a significant effect on the difference between the two groups. Species without significant differences are uniformly colored yellow, and red nodes indicate gut microbiota with significant differences

By analyzing the gut microbiota difference of the two groups, a total of 15 differential bacteria were screened. For example, the abundance of *Streptococcus, Enterococcus* and *Ruminococcus_gnavus_group* in CRC were increased compared with healthy individuals (Fig. 2A). LEfSe analysis showed 69 characteristic bacteria in the healthy group (*Clostridia, Oscillospirales, Ruminococcaceae, *etc.) and 85 characteristic bacteria in the CRC group (*Lactobacllales, Bacilli, Enterococcus, *etc.) (Fig. 2B and C)*.*

#### Correlation of differential bacteria between CRC and healthy group

**Fig. 3:**
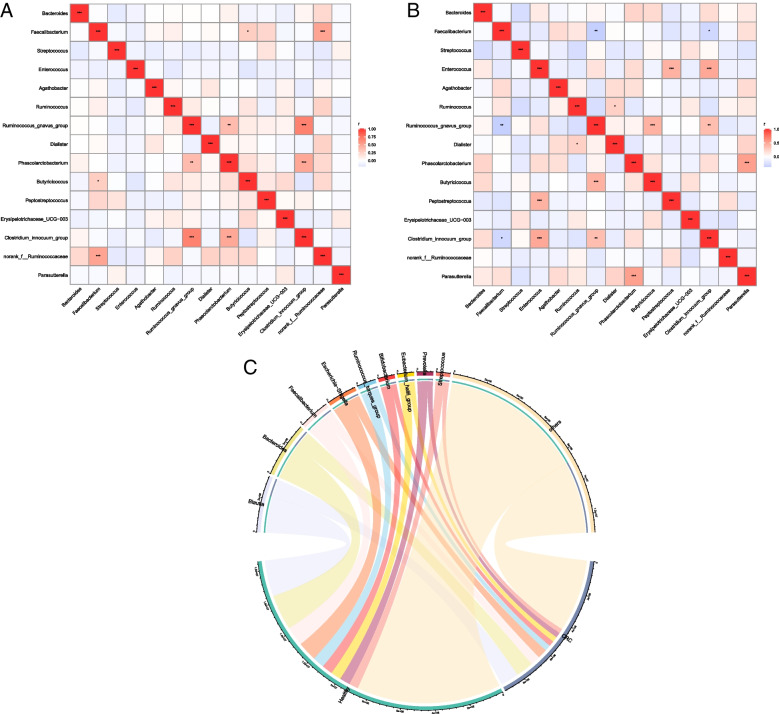
Correlation analysis of different gut microbiota in CRC and healthy groups. **A**, **B** Numerical matrices of the two different groups of bacteria were plotted using heat maps. Shades of color represent relevance. The redder the blocks in the figure, the more correlated the two bacterium are. Pearson's coefficient was used to calculate the correlation between bacterium. The shade of the color indicates the size of the data value. Pearson's correlation coefficients are shown in the figure (* 0.01 < *p* < 0.05, * * 0.001 < *p* ≤ 0.01, * * * *p* ≤ 0.001). **C** On one side of the circle are the names of gut bacteria, and the other side are the names of sample group. They are indicated in different colors, and species abundance is indicated in percentages

Gut microbiota was further analyzed by correlation analysis. In the CRC group, *Ruminococcus_gnavus* and *Clostridium_innocuum* were correlated (*r* = 0.679, *p* < 0.01), *Phascolarctobacteriu* and *Clostridium_innocuum* were correlated (*r* = 0.482, *p* < 0.001), and *Faecalibacterium* and *norank_f__Ruminococcaceae* were correlated (*r* = 0.508, *p* < 0.01). In the healthy group, *Enterococcus* and *Clostridium_innocuum* were correlated (*r* = 0.526, *p* < 0.01), *Enterococcus* and *Clostridium_innocuum* were correlated (*r* = 0.485, *p* < 0.001), and *Peptostreptococcus* and *Enterococcus* were correlated (*r* = 0.430, *p* < 0.001). The chord diagram showed that *Blautia* was more associated with the healthy group, rather than the CRC group (Fig. 3A-C).

#### Construction of predicting models for CRC

**Fig. 4:**
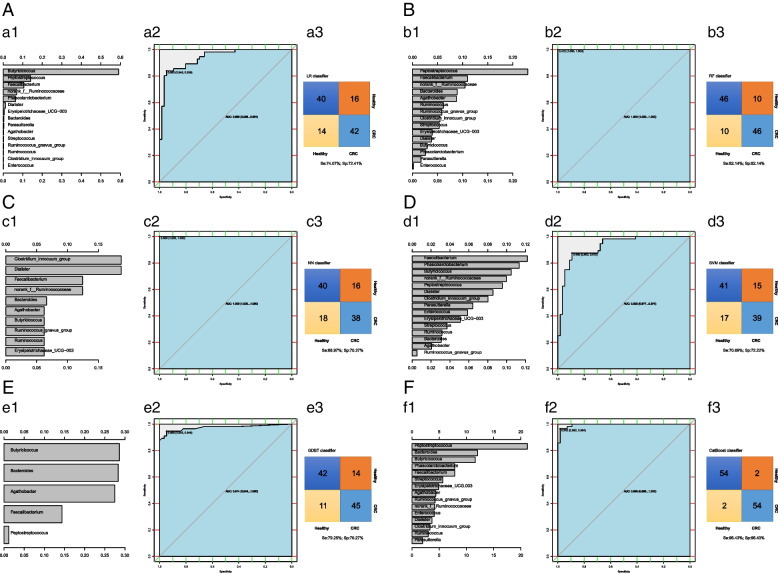
Predicting models for CRC basing on gut microbiota The panel **A**–**F** were conducted based on LR model, RF model, NN model, SVM model, gradient boosting tree model and CatBoost model relatively. The a1, b1, c1, d1, e1, and f1 show the variable importance histograms of the model, the a2, b2, c2, d2, e2, and f2 show the AUC curves of the model, and a3, b3, c3, d3, e3, and f3 show the sensitivity and specificity of the model

The characteristic bacteria screened by the LR model were *Butyricicoccus, Peptostreptococcus, Faecalibacterium,* etc., and the AUC was 0.938. The characteristic bacteria screened by the RF model were *Peptostreptococcus, Faecalibacterium, norank_f__Ruminococcaceae*, etc. and the AUC was 1.000. The characteristic bacteria selected by the NN model were *Clostridium_innocuum, Dialister, Faecalibacterium,* etc., and the AUC was 1.000. The characteristic bacteria selected by the SVM model were *Faecalibacterium, Phascolarctobacterium Phascolarctobacterium,* etc., and the AUC was 0.925. The characteristic bacteria selected by the GBDT model were *Butyricicoccus, Bacteroides, Agathobacter,* etc., and the AUC was 0.974. The characteristic bacteria screened by the CatBoost model were *Peptostreptococcu, Butyricicoccus, and Butyricicoccus*, and the AUC was 0.988. It can be seen that the best model for predicting CRC was the RF model. Overall, the accuracy of CatBoost model was higher (Se:96.43%, Sp:96.43%) (Fig. 4).

### Comparative analysis of hypoleukocytes and normal leukocytes after chemotherapy in patients with CRC

#### Structure and diversity of gut microbiota in patients with hypoleukocytes and normal leukocytes after chemotherapy for CRC

**Fig. 5:**
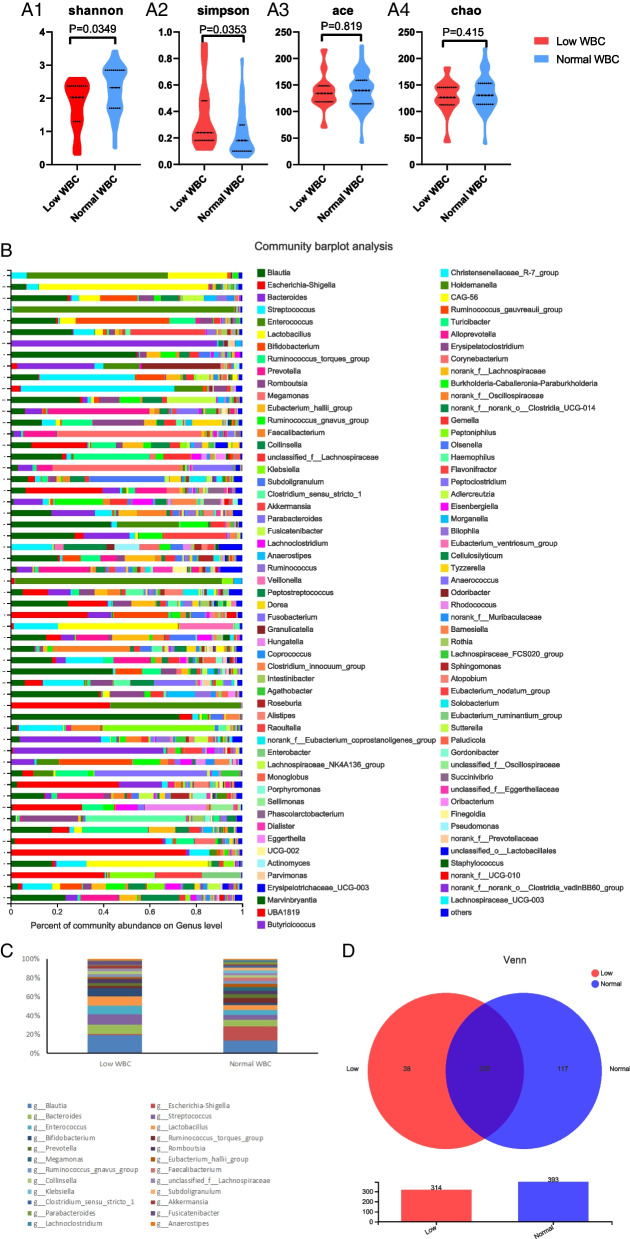
Descriptive analysis between hypoleukocytes and normal leukocytes group after CRC chemotherapy. **A** Alpha diversity was used to show species abundance of gut bacteria, with A1, A2, A3 and A4 showing Shannon index, Simpson index, Ace index and Chao index, respectively. **B** The gut bacteria composition between the groups was plotted. The ordinate is the group name, and the abscissa represents the proportion of bacteria in the sample. Different colors represent different bacterial groups, and the length of the column represents the proportion of bacterial groups. **C** Histograms of the cumulative percentages of the top 30 most abundant bacteria in both groups were plotted. **D** Red represents the hypoleukocytes group after chemotherapy, blue refers to the normal leukocytes group after chemotherapy, and the overlapping part stands for the number of common bacteria in the two groups. Below is a Venn diagram of the total gut bacteria of the two groups at the genus level

Compared with hypoleukocytes and normal leukocytes in CRC patients after chemotherapy, the community diversity of hypoleukocytes group was significantly decreased (*p* < 0.05), and there was no difference in abundance (*p* > 0.05) (Fig. 5 A1–A4). The gut microbiota community structure was different between the two groups (Fig. 5B). The top five bacteria with the highest composition ratio in both groups were *Blautia, Escherichia-Shigela, Streptococcus, Bacteroides* and *Enterococcus*. In the hypoleukocytes group, the proportion of *Blautia* was higher and the proportion of *Escherichia Shigella* was lower (Fig. 5C)*.* The Venn diagram showed 276 common bacteria of both groups, with 117 unique bacteria to the normal leukocytes group and 38 unique bacteria to the hypoleukocytes group (Fig. 5D). The sequencing depth was shown in Table 4.

**Table 4:** Diversity index table about low WBC and normal WBC after chemotherapy for CRC. chao and ace were the indices of community richness. shannon index, simpson index and coverage index were the indices of community diversity

Groups	shannon	simpson	ace	chao	coverage
Low WBC Group	2.613	0.113	117.006	111.176	1.000
	1.429	0.460	136.888	129.882	0.999
	1.158	0.443	69.793	42.429	0.998
	2.407	0.218	148.816	144.438	0.999
	1.348	0.544	143.298	145.250	0.999
	2.517	0.121	116.788	113.067	1.000
	1.789	0.256	217.289	183.048	0.999
	0.302	0.916	131.685	98.500	0.999
	2.272	0.210	122.481	121.111	1.000
	2.352	0.160	118.794	122.333	0.999
	2.007	0.312	124.235	156.200	0.999
	2.362	0.189	148.510	146.688	1.000
	2.053	0.225	182.335	144.750	0.999
	0.560	0.796	138.563	123.000	0.999
Normal WBC Group	2.892	0.101	150.101	142.667	0.999
	1.631	0.327	177.618	132.000	0.999
	2.489	0.145	118.538	117.583	1.000
	2.926	0.130	187.244	199.273	0.999
	1.641	0.412	93.555	92.000	1.000
	2.844	0.094	204.636	185.500	0.999
	2.719	0.101	102.252	98.231	1.000
	2.767	0.121	135.213	133.273	1.000
	1.879	0.290	171.260	141.882	0.999
	2.704	0.098	115.230	109.059	1.000
	1.837	0.277	111.264	125.429	1.000
	1.316	0.358	96.575	100.857	1.000
	0.760	0.499	108.416	102.600	0.999
	2.927	0.137	177.487	183.000	1.000
	2.539	0.142	140.536	146.111	1.000
	2.318	0.184	125.058	124.100	0.999
	1.706	0.432	103.358	109.667	1.000
	2.328	0.222	132.955	126.833	1.000
	2.726	0.103	130.754	128.615	1.000
	1.215	0.542	121.735	97.250	1.000
	2.804	0.098	111.492	113.667	1.000
	2.316	0.236	154.455	152.500	0.999
	1.691	0.343	144.100	139.200	1.000
	1.951	0.201	149.006	155.200	0.999
	2.894	0.144	155.083	151.563	1.000
	3.364	0.056	180.364	186.000	0.999
	2.990	0.088	148.048	152.111	1.000
	1.534	0.268	115.092	124.100	0.999
	3.112	0.063	138.870	141.100	1.000
	3.089	0.085	224.456	219.500	0.999
	2.304	0.179	124.050	118.769	0.999
	2.134	0.253	152.615	151.500	1.000
	2.799	0.115	170.609	163.556	0.999
	2.229	0.194	116.374	113.545	1.000
	0.496	0.800	41.150	39.200	1.000
	1.603	0.425	107.759	112.857	1.000
	2.046	0.190	148.378	122.000	1.000
	1.193	0.578	113.560	115.100	1.000
	1.772	0.277	150.280	129.083	0.999
	3.435	0.073	177.425	180.000	0.999
	2.863	0.098	149.121	157.111	0.999
	2.677	0.149	174.768	163.550	0.999

### Difference of gut microbiota between hypoleukocytes and normal leukocytes group

**Fig. 6:**
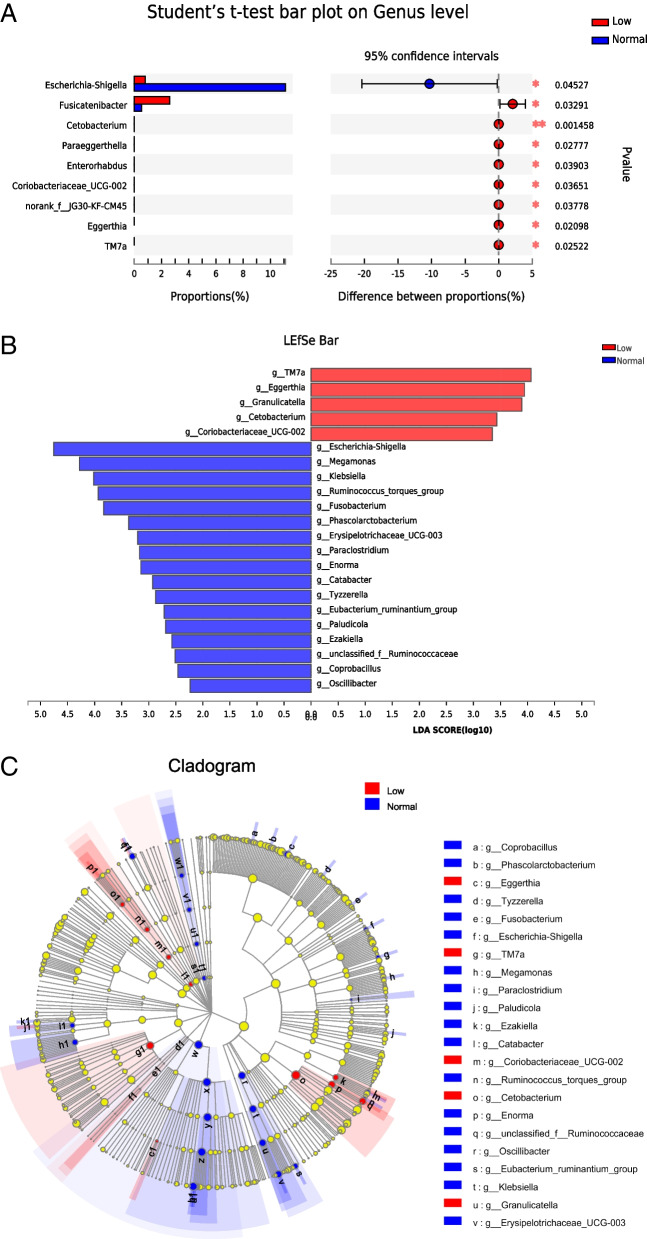
Difference analysis between hypoleukocytes and normal leukocytes group after CRC chemotherapy. **A** The t-test method was used to test the hypothesis of the gut microbiota of the two groups and evaluate the significance level of the difference in the abundance of the bacteria. *P*<0.05 was considered statistically significant. **B** LDA was used to draw histograms and count the gut microbiota with significant differences between the two groups. LDA scores were obtained using linear regression analysis. The greater the score, the greater the influence of gut microbiota abundance on differential effects. **C** From inner circle to outer circle, the bacteria at different levels of phylum, class, order, family, genus, and species are represented in turn. Different colored nodes indicate the degree of enrichment of bacteria in the corresponding group and whether they have a significant effect on the difference between groups. Species with no significant differences are uniformly colored yellow, and red nodes indicate gut microbiota with significant differences

After analyzing the gut microbiota difference of the two groups, 9 differential bacteria were screened. In instance, the abundance of *Fusicatenibacter, Cetobacterium* and *Paraeggerthella* in CRC with hypoleukocyte compared with normal CRC (Fig. 6A). LEfSe analysis showed 5 differential bacteria in the hypoleukocytes group including *Eggerthia, Granulicatella*, *Cetobacterium* and 17 differential bacteria in the normal leukocytes group including *Escherichia-Shigella, Megamonas, Klebsiella* (Fig. 6B and C)*.*

### Correlation of differential bacteria between hypoleukocytes and normal leukocytes group

**Fig. 7:**
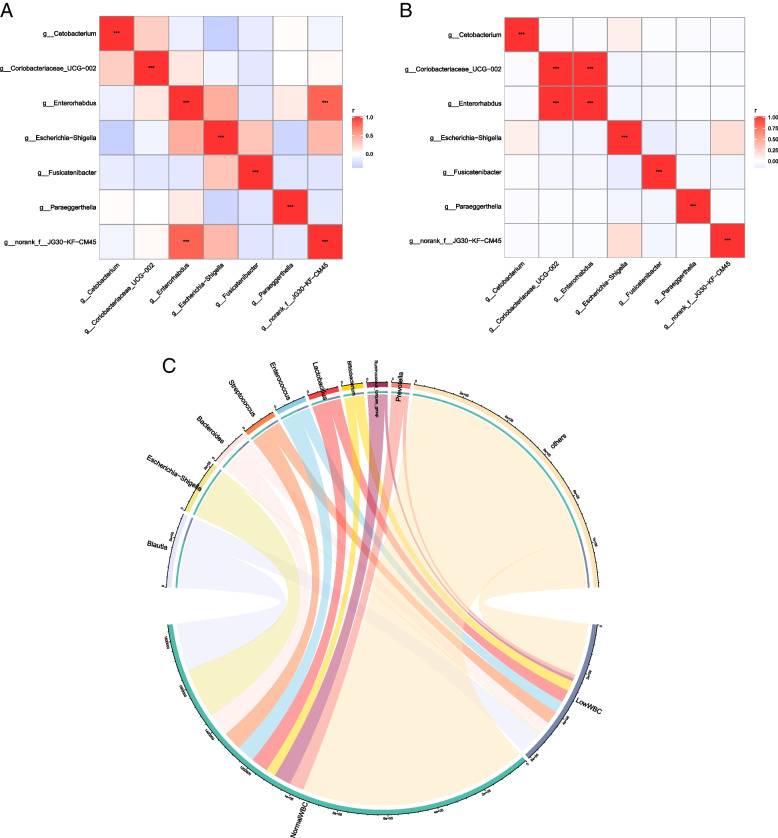
Correlation analysis of different gut microbiota in hypoleukocytes and normal leukocytes groups after CRC chemotherapy. **A**, **B** Numerical matrices of two different groups of bacteria were plotted using heat maps. The shades of color represent relevance. The redder the blocks in the figure, the more correlated the two bacterium are. Pearson's correlation coefficient was used to calculate the correlation between bacterium. The shade of the color indicates the size of the data value. Pearson's correlation coefficients are shown in the figure (* 0.01 < *p* < 0.05, * * 0.001 < *p*≤0.01, * * * *p*≤0.001). **C** On one side of the circle are names of gut bacteria, and the other side are names of sample group. They are indicated by different colors, and species abundance is indicated by percentages.

The gut microbiota was further analyzed by correlation chart analysis. In the hypoleukocytes group, *Enterorhabdus* and *norank-f-JG30-KF-CM45* were correlated (*r* = 0.826, *p* < 0.001). In the normal leukocytes group, *Coriobacteriaceae_UCG-002* and *Enterorhabdus* were correlated (*r* = 1, *p* < 0.001). The chord diagram showed that *Escherichia-Shigella* was more strongly related to the normal leukocytes group, rather than the hypoleukocytes group (Fig. 7A–C).

### Construction of predicting models for leukopenia after chemotherapy for CRC

**Fig. 8:**
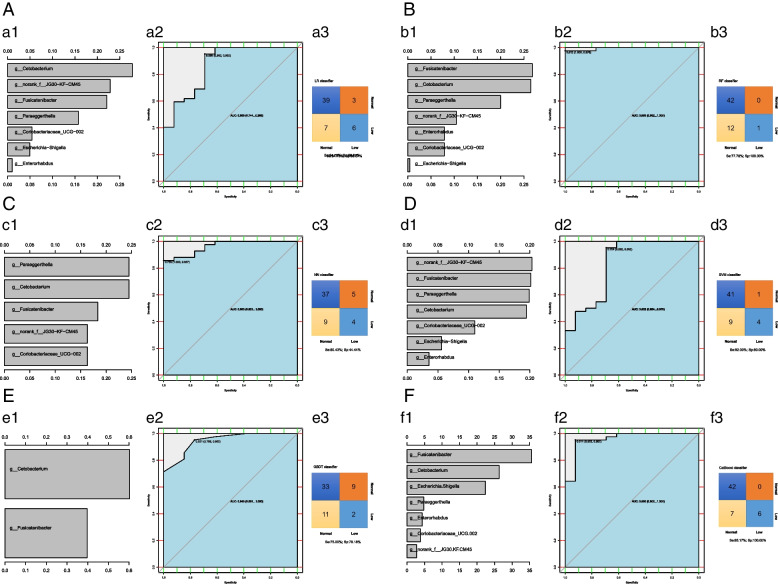
Predicting models for CRC with hypoleukocytes after chemotherapy basing on gut microbiota The panel **A**–**F** were conducted based on LR model, RF model, NN model, SVM model, gradient boosting tree model and CatBoost model relatively. The a1, b1, c1, d1, e1, and f1 show the variable importance histograms of the model, the a2, b2, c2, d2, e2, and f2 show the AUC curves of the model, and a3, b3, c3, d3, e3, and f3 show the sensitivity and specificity of the model

The characteristic bacteria selected by the LR model were *Cetobacterium, norank_f__JG30-KF-CM45, Fusicatenibacter*, etc., and the AUC was 0.866. The characteristic bacteria screened by the RF model were *Fusicatenibacte, Cetobacterium, Paraeggerthella*, etc., and the AUC was 0.995. The characteristic bacteria selected by the NN model were *Paraeggerthella, Cetobacterium, Fusicatenibacte*, etc., and the AUC was 0.963. The characteristic bacteria selected by the SVM model were *norank_f__JG30-KF-CM45, Fusicatenibacter, Paraeggerthella*, etc., and the AUC was 0.832. The characteristic bacteria selected by the GBDT model were *Cetobacterium* and *Fusicatenibacte,* and the AUC was 0.948. The characteristic bacteria screened by the CatBoost model were *Fusicatenibacter, Cetobacterium, Escherichia-Shigella,* etc., and the AUC was 0.960. It can be seen that the best model for predicting the gut microbiota of leukopenia after chemotherapy for CRC was RF model. On the whole, the accuracy of CatBoost model was higher (Se:85.17%, Sp:100%) (Fig. 8).

## Discussion

The study of gut microbiota and leukopenia after chemotherapy has received increasing attention, and determining the specific link between the two remains a challenge. In the present research, fecal samples were collected from 56 healthy people and 55 chemotherapy patients with CRC. The top 5 bacteria with the highest constituent ratio in the healthy group and CRC group were *Blautia, Escherichia Shigella, Bacteroides, Streptococcus* and *Faecalibacterium*. The community structure and alpha diversity of gut microbiota in CRC group decreased significantly. Moreover, chemotherapy can decrease the number of WBC and result in a decrease in the community structure and alpha diversity of the microbiome community structure. The present study provides a new direction for the exploration of chemotherapy-induced leukopenia in CRC from the perspective of gut microbiota. After predicting that patients are at high risk for leukocyte decline after chemotherapy we can take a number of measures to prevent this side effect. For example, patients can be treated with leukocytotropic drugs to reduce the occurrence or severity of myelosuppression at the same time of chemotherapy. Reducing the dose of chemotherapy or delaying chemotherapy may also be a good option. Of course, increasing the frequency of follow-up after chemotherapy to achieve dynamic detection of white blood cell levels will also help the treatment of myelosuppression and the recovery of patients.

The mechanism of gut microbiota on CRC is related to the increased production of toxins by bacteria, decreased production of metabolites derived from beneficial bacteria, disruption of the epithelial barrier, production of pro-cancer compounds, or changes in the gut microbiota [24]. The correlation between intestinal microbial community structure and CRC has been confirmed by more and more studies [12, 13, 25], which is also reflected in this study. For example, *Streptococcus* and *Enterococcus faecalis*, which were more in CRC compared with healthy individuals in this result, are potential pathogen of CRC [26, 27]. Being consistent with most previous findings [28], gut microbiota diversity and abundance were significantly decreased in CRC patients in the study. Our results once again validate the correlation between CRC and gut microbiota from a clinical perspective. In the future, we may further investigate the mechanisms by which these gut microbiota promote or inhibit CRC.

Subsequently, in this study, by further analyzing the alterations in the gut microbiota of the WBC decrease in CRC after chemotherapy, there was no difference in the abundance of colony species between the hypoleukocytes group and normal leukocytes group, while the community diversity was remarkably reduced. Past studies at this level have been unclear, but it is interesting to note that there are also differences in gut microbiota in people who are effective and ineffective at CRC chemotherapy, and even *Roseburia* can be a predictor for them (Se:75.0%;Sp:93.9%) [29]. In this study, based on a variety of machine learning algorithms, a model was constructed to predict whether there was a decrease in WBC after chemotherapy, and CatBoost model was found to have the highest accuracy (Se:85.17%, Sp:100%). The *Fusicatenibacter, Cetobacterium, Escherichia-Shigella, *etc. have played an important role in the prediction. *Fusicatenibacter* has also previously been reported to be associated with chemotherapy-induced diarrhea [30]. Escherichia-Shigella is also an important bacteria involved in inflammation of gut [31]. Studies on *Cetobacterium* and CRC or chemotherapy have not been clearly reported. Moreover, we have not been able to determine whether these bacterial changes are a cause or a consequence of the decrease in WBC after chemotherapy. In the future, we will continue to complete animal studies to clear the mechanism between these bacteria and the decrease of WBC after chemotherapy.

Representative differential bacteria such as *Bacteroides, Faecalibacterium* and *Streptococcus* were screened from healthy individuals and CRC patients in this study. Others have shown that the bacteria that are most associated with CRC are *Clostridium nucleatum, Streptococcus digestiveis,* and *Bacteroides fragilis* [32]. A meta-analysis of four case–control studies of the macrogenome of CRC patients found that *Bacteroides fragilis* was the only species and consistently enriched in the gut microbiota of CRC patients worldwide [33]. In addition, a study found the correlation between *Enterococcus* and CRC. The bacterium is more highly aggregated in fecal specimens from CRC patients compared with healthy controls, and more abundant in the adjacent tissues of cancer and CRC compared with healthy mucosa [34]. In this study, *Escherichia-Shigella* was also found to be more strongly correlated with the post-chemotherapy WBC normal group compared with the post-chemotherapy hypoleukocytes group. It was inferred that there may be a link between WBC and gut microbiota. In this regard, it is recommended that patients' WBC levels should be measured after chemotherapy, and the association between reduced WBC levels and gut microbiota should be further analyzed. Overall, the association of gut microbiota with CRC or leukopenia after chemotherapy is well established, but we may need to confirm this with larger samples.

In the future, monitoring WBC and gut microbiota changes after chemotherapy in CRC may help detect and intervene disease and promote the development of disease prevention methods. The potential of gut microbiota, as a CRC biomarker, may provide new ideas for the use of gut microbiota to prevent and treat CRC and prevent leukopenia after chemotherapy in the future. Some gut microbiota agents that may accompany chemotherapy will be developed to reduce myelosuppression after chemotherapy. However, there are still some shortcomings in the current study. First, due to the limited sample size, this study could not carry out more subgroup analysis and the confidence of the results is uncertain. Subsequent studies can further carry out subgroup analysis of chemotherapy-induced colitis, malnutrition and other diseases. Second, the samples of healthy people kept at our hospital are relatively young in this study. People over 45 years old have a high incidence of CRC, so the original designers of this study included people over 45 years old. However, the majority of people who actually received chemotherapy during this period were patients with middle to later stage CRC, and this segment of patients was generally older to induce a certain difference in age between the control group and the experimental group. In addition, we included patients as samples from a time period in the real world, and we didn't screen too harshly for age, sex and drinking history. In the future, we will make more stringent requirements on this aspect to explore deeper correlations between gut microbiota and myelosuppression after chemotherapy. Finallly, the causal relationship between altered gut microbiota and CRC development remains unclear. Due to the uncertainty of causality, there are still some challenges in truly applying gut microbiota to clinical practice. Therefore, a larger sample size and further mechanism study are needed for further verification to provide data support for future clinical application.

### Supplementary Information

**Additional file 1.**

**Additional file 2.**

**Additional file 3.**

## Data Availability

The datasets generated for this study can be accessed from the NCBI Sequence Read Archive (SRA) database under the accession number PRJNA905191(http://www.ncbi.nlm.nih.gov/bioproject/905191). The data has been released to the public.

## Abbreviations

CRC: Colorectal cancer
AJCC: American Joint Committee on Cancer
LDA: Linear discriminant analysis
LR: Logistic regression
RF: Random forest
NN: Neural network
SVM: Support vector machine
GBDT: Gradient boosting decision tree
KW: Kruskal–Wallis
CIGT: Chemotherapy-induced gastrointestinal toxicity
WBC: White blood cell
St test: Student's t-test
