# Supplementary material for: Correlation of gut microbiota with leukopenia after chemotherapy in patients with colorectal cancer
Source: BMC Microbiol. 2023 Nov 17;23:349. doi: 10.1186/s12866-023-03067-6 (PMC10655349; doi:10.1186/s12866-023-03067-6)
Supplement: Supplementary file 1 — Additional file 1. [file 12866_2023_3067_MOESM1_ESM.pdf]

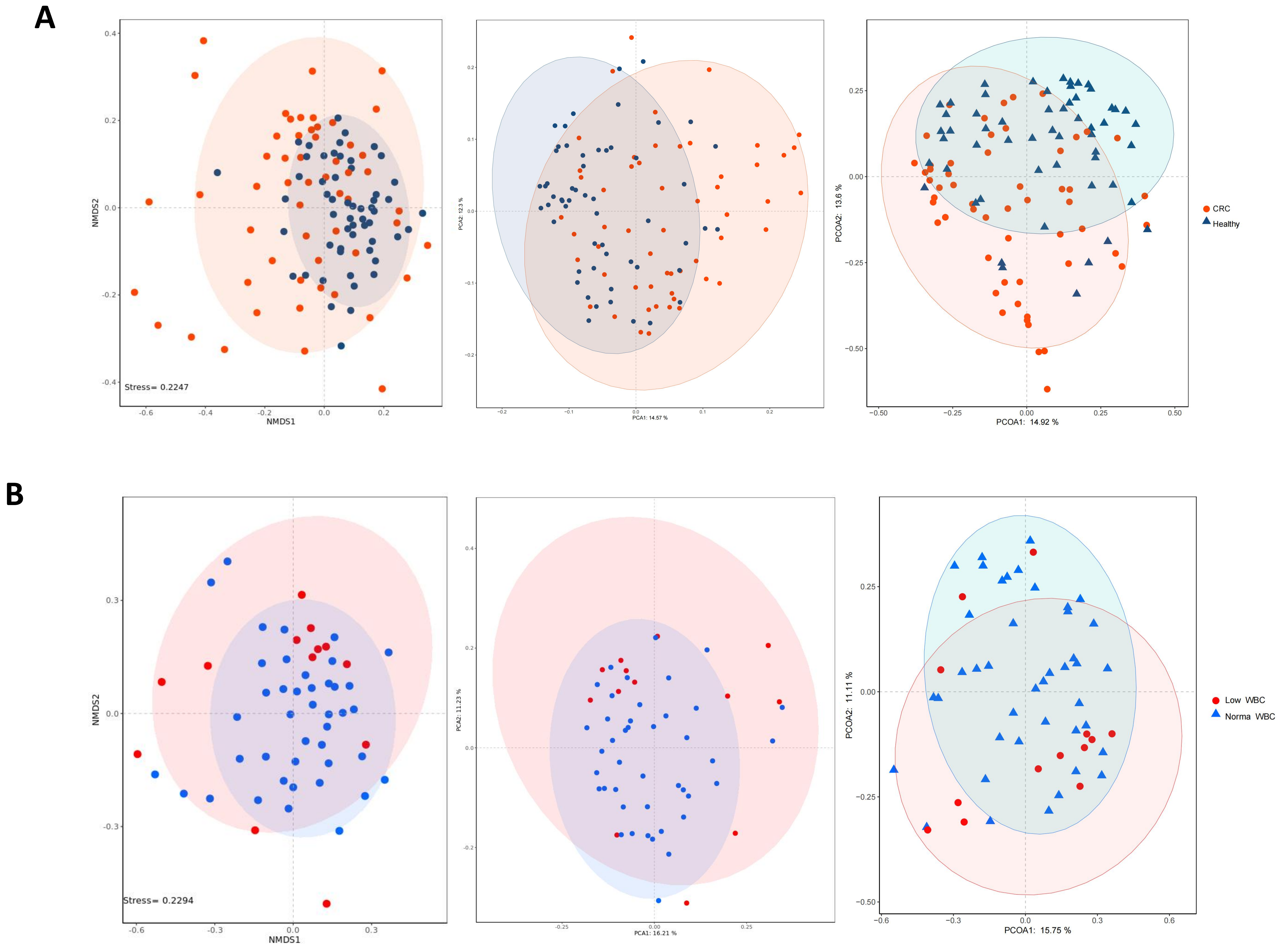

Supplementary File 1 Legend

The beta diversity was used to show species distribution of gut bacteria, including NMDS, PCA and PCoA. The panel A was the distribution of CRC patients and healthy individuals. The panel B was the distribution of hypoleukocytes and normal leukocytes group after CRC chemotherapy
