# Supplementary material for: Correlation of gut microbiota with leukopenia after chemotherapy in patients with colorectal cancer
Source: BMC Microbiol. 2023 Nov 17;23:349. doi: 10.1186/s12866-023-03067-6 (PMC10655349; doi:10.1186/s12866-023-03067-6)
Supplement: Supplementary file 2 — Additional file 2. [file 12866_2023_3067_MOESM2_ESM.pdf]

Supplementary File 2 The pathological results of CRC patients

Sample	Pathological type
CRC-1	Elevated moderately differentiated adenocarcinoma
CRC-2	Infiltrating moderately differentiated adenocarcinoma
CRC-3	Infiltrating meso-poorly differentiated adenocarcinoma with lymph node metastasis4/14
CRC-4	Elevated moderately differentiated adenocarcinoma
CRC-5	Ulcerative moderately differentiated adenocarcinoma with lymph node metastasis1/29
CRC-6	Elevated moderately differentiated adenocarcinoma
CRC-7	Ulcerative moderately differentiated adenocarcinoma
CRC-8	Elevated moderately differentiated adenocarcinoma
CRC-9	Ulcerative moderately differentiated adenocarcinoma
CRC-10	Elevated meso-poorly differentiated adenocarcinoma
CRC-11	Ulcerative moderately differentiated adenocarcinoma
CRC-12	Elevated moderately differentiated adenocarcinoma
CRC-13	Elevated moderately differentiated adenocarcinoma
CRC-14	Ulcerative moderately differentiated adenocarcinoma
CRC-15	Elevated moderately differentiated adenocarcinoma
CRC-16	Elevated moderately differentiated adenocarcinoma
CRC-17	Elevated moderately differentiated adenocarcinoma
CRC-18	Ulcerative moderately differentiated adenocarcinoma
CRC-19	Elevated moderately differentiated adenocarcinoma with lymph node metastasis 2/28
CRC-20	Elevated moderately differentiated adenocarcinoma
CRC-21	Ulcerative moderately differentiated adenocarcinoma
CRC-22	Elevated moderately differentiated adenocarcinoma
CRC-23	Elevated moderately differentiated adenocarcinoma with lymph node metastasis 5/22
CRC-24	Raised mucinous adenocarcinoma
CRC-25	Elevated moderately differentiated adenocarcinoma
CRC-26	Elevated meso-poorly differentiated adenocarcinoma with lymph node metastasis4/14
CRC-27	Elevated moderately differentiated adenocarcinoma
CRC-28	Elevated moderately differentiated adenocarcinoma
CRC-29	Ulcerative meso-poorly differentiated adenocarcinoma
CRC-30	Elevated moderately differentiated adenocarcinoma
CRC-31	Raised mucinous adenocarcinoma
CRC-32	Elevated moderately differentiated adenocarcinoma
CRC-33	Elevated meso-poorly differentiated adenocarcinoma with lymph node metastasis4/14
CRC-34	Elevated moderately differentiated adenocarcinoma
CRC-35	Elevated moderately differentiated adenocarcinoma
CRC-36	Elevated moderately differentiated adenocarcinoma
CRC-37	Elevated moderately differentiated adenocarcinoma
CRC-38	Elevated moderately differentiated adenocarcinoma
CRC-39	Elevated moderately differentiated adenocarcinoma
CRC-40	Elevated moderately differentiated adenocarcinoma with lymph node metastasis 2/28
CRC-41	Ulcerative moderately differentiated adenocarcinoma with lymph node metastasis 4/14
CRC-42	Ulcerative moderately differentiated adenocarcinoma
CRC-43	Ulcerative moderately differentiated adenocarcinoma
CRC-44	Elevated moderately differentiated adenocarcinoma
CRC-45	Ulcerative moderately differentiated adenocarcinoma
CRC-46	Undifferentiated eminence carcinoma
CRC-47	Ulcerative moderately differentiated adenocarcinoma
CRC-48	Elevated moderately differentiated adenocarcinoma
CRC-49	Ulcerative poorly differentiated adenocarcinoma
CRC-50	Ulcerative moderately differentiated adenocarcinoma
CRC-51	Ulcerative moderately differentiated adenocarcinoma with lymph node metastasis 2/11
CRC-52	Elevated moderately differentiated adenocarcinoma with lymph node metastasis 4/12
CRC-53	Ulcerative moderately differentiated adenocarcinoma
CRC-54	Ulcerative moderately differentiated adenocarcinoma
CRC-55	Elevated moderately differentiated adenocarcinoma
