# Supplementary material for: Correlation of gut microbiota with leukopenia after chemotherapy in patients with colorectal cancer
Source: BMC Microbiol. 2023 Nov 17;23:349. doi: 10.1186/s12866-023-03067-6 (PMC10655349; doi:10.1186/s12866-023-03067-6)
Supplement: Supplementary file 3 — Additional file 3. [file 12866_2023_3067_MOESM3_ESM.pdf]

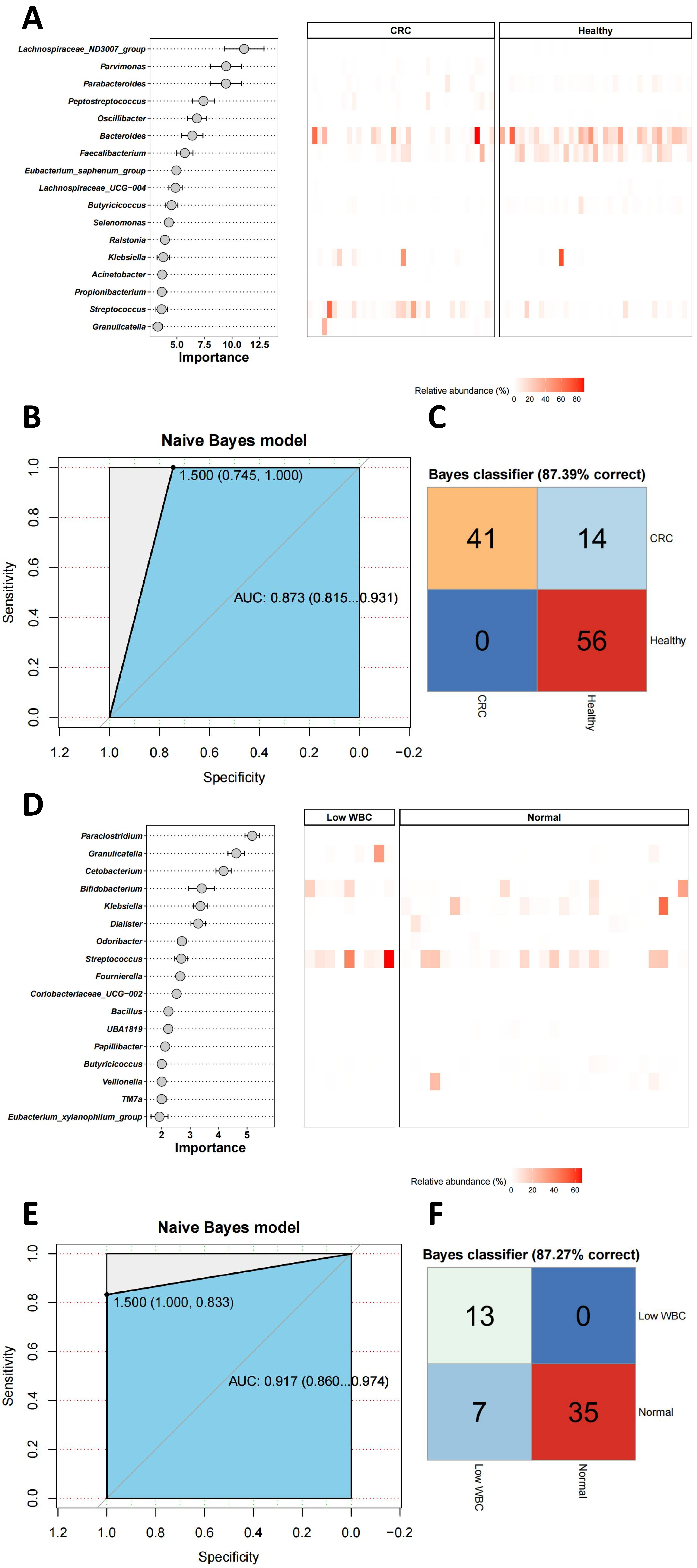

Supplementary File 3 Legend

The panel A-C were predicting models for CRC based on Naïve Bayes model and the panel D-F were the predicting models for CRC with hypoleukocytes after chemotherapy. The A and D show the variable importance histograms of the model, the B and E show the AUC curves of the model, and C and F show the sensitivity and specificity of the model.
